# Excessive Iron Induces Oxidative Stress Promoting Cellular Perturbations and Insulin Secretory Dysfunction in MIN6 Beta Cells

**DOI:** 10.3390/cells10051141

**Published:** 2021-05-09

**Authors:** Voni Blesia, Vinood B. Patel, Hisham Al-Obaidi, Derek Renshaw, Mohammed Gulrez Zariwala

**Affiliations:** 1 Centre for Nutraceuticals, School of Life Sciences, University of Westminster, 115 New Cavendish Street, London W1W 6UW, UK; w1564070@my.westminster.ac.uk (V.B.); v.b.patel@westminster.ac.uk (V.B.P.); 2 The School of Pharmacy, University of Reading, Whiteknights, P.O. Box 226, Reading RG6 6AP, UK; h.al-obaidi@reading.ac.uk; 3 Centre for Sport, Exercise and Life Sciences, Institute of Health & Wellbeing, Coventry University, Priory St, Coventry CV1 5FB, UK; derek.renshaw@coventry.ac.uk

**Keywords:** excess iron, oxidative stress, impaired insulin secretion, type 2 diabetes mellitus, β-cell

## Abstract

Exposure to high levels of glucose and iron are co-related to reactive oxygen species (ROS) generation and dysregulation of insulin synthesis and secretion, although the precise mechanisms are not well clarified. The focus of this study was to examine the consequences of exposure to high iron levels on MIN6 β-cells. MIN6 pseudoislets were exposed to 20 µM (control) or 100 µM (high) iron at predefined glucose levels (5.5 mM and 11 mM) at various time points (3, 24, 48, and 72 h). Total iron content was estimated by a colourimetric FerroZine™ assay in presence or absence of transferrin-bound iron. Cell viability was assessed by a resazurin dye-based assay, and ROS-mediated cellular oxidative stress was assessed by estimating malondialdehyde levels. β-cell iron absorption was determined by a ferritin immunoassay. Cellular insulin release and content was measured by an insulin immunoassay. Expression of SNAP-25, a key protein in the core SNARE complex that modulates vesicle exocytosis, was measured by immunoblotting. Our results demonstrate that exposure to high iron levels resulted in a 15-fold (48 h) and 4-fold (72 h) increase in cellular iron accumulation. These observations were consistent with data from oxidative stress analysis which demonstrated 2.7-fold higher levels of lipid peroxidation. Furthermore, exposure to supraphysiological (11 mM) levels of glucose and high iron (100 µM) at 72 h exerted the most detrimental effect on the MIN6 β-cell viability. The effect of high iron exposure on total cellular iron content was identical in the presence or absence of transferrin. High iron exposure (100 µM) resulted in a decrease of MIN6 insulin secretion (64% reduction) as well as cellular insulin content (10% reduction). Finally, a significant reduction in MIN6 β-cell SNAP-25 protein expression was evident at 48 h upon exposure to 100 µM iron. Our data suggest that exposure to high iron and glucose concentrations results in cellular oxidative damage and may initiate insulin secretory dysfunction in pancreatic β-cells by modulation of the exocytotic machinery.

## 1. Introduction

Type 2 diabetes mellitus (T2DM), also known as insulin-dependent diabetes mellitus (IDDM), is a global health problem with a rapidly increasing incidence. As per the International Diabetes Foundation (IDF), there were more than 387 million people affected in 2014, and with numbers increasing every year this is projected to reach 592 million by 2035 [1]. T2DM represents a serious public health concern that is prevalent in both developed and developing countries [2].

Insulin is a key homeostatic regulator of blood glucose. Defects in both insulin secretion and insulin action contribute to the development of T2DM [3]. Synaptosomal associated protein 25 (SNAP-25) is one of core components of SNARE (soluble N-ethylmaleimide-sensitive factor attachment protein receptor) proteins and contains amino acid and carboxy terminals that bind to different proteins [4]. SNAP-25 binds to syntaxin 1 and VAMP2 and together the three SNARE proteins generate a steady bundle of the four α-helices, with each protein containing one α-helix (from VAMP and syntaxin), and two α-helices from SNAP23/25 [4]. These proteins have a crucial role in regulating = vesicle/granule exocytosis of not only insulin, but also sharing numerous commonalities with neuronal synaptic vesicle exocytosis [4]. Pancreatic β-cells have a crucial role to synthesise and secrete insulin at appropriate rates. However, these cells are susceptible to oxidative damage [5], as they have relatively lower expression of protective antioxidant enzymes, such as catalase and glutathione peroxidase compared to other organs [6]. At low concentrations, reactive oxygen species (ROS) provide beneficial effects in many metabolic processes [7]. Unfortunately, these powerful agents can be important mediators of damage to cell structures, nucleic acids, lipids, and proteins, that is associated with biochemical and functional changes [8]. Lipid peroxidation is one biological process resulting from the attack by metal-induced generation of ROS, producing malondialdehyde (MDA) and 4-hydroxy-2-nonenal (4-HNE) [9].

As a consequence of exposure to high concentrations of ROS, pancreatic β-cell can experience oxidative stress, resulting in cellular toxicity [10]. Recent studies have shown that iron overaccumulation in the β-cell leads to increased mitochondrial iron transport, which induces mitochondrial dysfunction [11]. Iron overload contributes to various complex diseases, including diabetes mellitus [12] and neurodegenerative diseases such as Alzheimer’s disease and Parkinson’s disease [13]. 

Iron is the most important trace element and is found in many essential enzymes and proteins. This transition metal exists in two valency states, Fe^2+^ (ferrous iron) and Fe^3+^ (ferric iron) and has various essential functional roles in the body including a vital contribution to the redox reactions of oxidative phosphorylation by accepting and donating electrons [14]. Unfortunately, the same properties that make iron useful can also make it toxic to cells. Through the generation of free radicals, iron can damage essential biological components such as DNA, proteins, and lipids [15]. Thus, an organism must have control over iron uptake, and its location and transport within the body. When the amount of free ferrous iron is elevated, it is believed to lead to the generation of ROS through Fenton and Haber-Weiss chemistry [16]. The hydroxyl radical (•OH) that is produced via Fenton chemistry is the most reactive oxygen radical and can readily react with biological molecules in its immediate vicinity causing significant damage [17]. •OH is a major species that attacks cell membrane lipids, proteins, and DNA and causes tissue damage leading to insulin resistance and eventually β-cell failure [18].

As described above, ROS clearly possess the capacity to behave as a destructive agent, and the key element to prevent this toxicity is the induction of antioxidant defense systems. Unfortunately, chronic oxidative stress could lead to the progression of pancreatic β-cell dysfunction due to a lack of significant protective agents. It is therefore important to improve our understanding of the relationship between iron and ROS-mediated changes in β-cell function.

Thus, this study aimed to assess cellular oxidative dysfunction mediated by ROS in pancreatic β-cells and its effects upon cell viability, insulin synthesis, and secretion. The effect of β-cell oxidative stress on expression of SNAP-25, a key protein involved in the insulin exocytosis machinery, was also examined.

## 2. Materials and Methods

### 2.1. Materials

All chemicals were cell culture grade and purchased from Sigma-Aldrich (Poole, UK) and Thermo Fisher Scientific (Loughborough, UK) unless otherwise stated. Murine MIN6 cells were a kind gift from Dr. Bo Liu (Department of Diabetes, King’s College London, London, UK) at passage 34 (originally from Prof. Jun-ichi Miyazaki, Osaka University, Osaka, Japan). Thiobarbituric Acid Reactive Substances (TBARS) parameter kit (Cat No. KGE013) was from R&D Systems (Abington, UK). PrestoBlue^TM^ cell viability reagent was purchased from Thermo Fisher Scientific (Loughborough, UK, catalogue no. A13261). Ferritin ELISA kit (product code S-22) was from ATI Atlas (Chichester, UK). Mouse insulin ELISA was supplied by Mercodia (Cat No. 10-1247-01—Mercodia, Diagenics, Milton Keynes, UK). 

### 2.2. MIN6 Cell Culture

Murine MIN6 cells were prepared and obtained at passage 34–40 in complete Dulbecco’s Modified Eagle Medium (DMEM)—GlutaMax^®^, pH 7.4 supplemented with 15% Foetal Bovine Serum (FBS), 1% antibiotic/antimycotic solution and 25 mM HEPES. The cells were incubated in an atmosphere of 95% air and 5% CO_2_ at constant humidity. Stock cultures were grown at 37 °C in 75 cm^2^ T-flasks, replacing the medium every two days. Cells were seeded in 12-well plates with density of 25 × 10^4^ cells/cm^2^ for all experimental cultures. MIN6 cells reached confluence within day 3–5 post-seeding by which point the phenotype of small clusters of cells was formed (Supplementary Figure S1), indicating the cells were functional and capable of secreting insulin [19].

### 2.3. MIN6 Cell Exposure to Glucose and Iron

The glucose and iron exposure experiment was carried out using serum-free MEM with 5.5 mM glucose. Media were supplemented with 2 mM L-glutamine, 1% antibiotic/antimycotic, 0.1% BSA, and 25 mM HEPES, pH 7.4. Cells were seeded at 25 × 10^4^ cells/cm^2^. The cells were iron depleted with serum-free media treatment and incubated overnight prior to conducting the experiment at 37 °C with 5% CO_2_. MIN6 cells were preincubated for 1 h in Krebs–Ringer Bicarbonate (KRB) buffer (119 mM NaCl, 4.74 mM KCl, 2.54 mM CaCl·6H_2_O, 1.19 mM KH_2_PO_4_, 1.19 mM MgSO_4_·7H_2_O, 25 mM NaHCO_3_, 10 mM HEPES, pH 7.4, and 1% BSA) containing 1.1 mM glucose (basal) before the glucose and iron stimulation.

MIN6 cells were washed with DPBS and placed in KRB buffer containing varying concentrations of glucose (5.5 mM (physiological) and 11 mM (supraphysiological), and iron [20 μM (control) and 100 μM (high)], or a combination of both glucose and iron, with an addition of tolbutamide (100 μM) (Table 1). Preincubation was carried out at 3, 24, 48, and 72 h time intervals. Cells then were lysed with RIPA buffer with the addition of protease inhibitor cocktail (PIC) and then subsequently stored at −20 °C.

### 2.4. Intracellular Total Iron Quantification

Intracellular total iron levels were quantified using an optimised FerroZine™-based iron assay according to method developed by Reimer et al. [20]. Iron detection reagent was added into each standard and sample tube containing 6.5 mM FerroZine™, 6.5 mM neocuproine, 2.5 M ammonium acetate, and 1 M ascorbic acid dissolved in water. The standard or sample mixtures with reagents underwent incubation for 30 min at room temperature. Following incubation, colour development was observed and 280 μL from both standard and sample tubes was added in duplicate into wells of a 96-well plate before the absorbance was measured at 550 nm using a microplate reader. Intracellular iron concentration was determined by normalising against a total protein standard curve which was obtained using the BCA assay.

### 2.5. Intracellular Ferritin Quantification

Ferritin content of MIN6 cells was determined using a commercial ELISA kit according to the manufacturer’s instructions. This experiment was followed by measuring the total protein content of MIN6 cells using a Pierce™ BCA kit (Thermo Fisher Scientific, Loughborough, UK, Cat No. 23225). The kit-provided Bovine Serum Albumin (BSA) was used as a stock (2 mg/mL) to prepare standards by serial dilutions. Samples were loaded in duplicate into a 96-well plate (25 μL). Absorbance was measured at 490 nm with background correction at 630 nm using a microplate reader. Ferritin concentration was standardised against the total protein concentration determined by the BCA assay.

### 2.6. Lipid Peroxidation Assessment

The samples from the static incubation experiments stored at −20 °C were assessed for lipid peroxidation using a TBARS assay. Manufacturer’s protocol was followed with minor variations. A stock solution of 167 μM was prepared with a combination of 100 μL TBARS Standard and 200 μL TBARS Acid Reagent, kept at RT for 30 min with gentle agitation at 30 RPM. TBARS Acid and TBA Reagent were added on each designated sample and placed in a water bath at 80 °C for 1.5 h. The absorbance was measured at 532 nm and followed by subtracting the pre-reading from the final reading.

### 2.7. Measurement of Cellular Cytotoxicity

Cells were prepared as described above and treated with iron/glucose for 3, 24, 48, and 72 h. PrestoBlue^TM^ cell viability reagent was performed following manufacturer’s protocol (1 h incubation) to measure cell cytotoxicity. Absorbance was measured at 560–590 nm and absorbance measures were compared to control cells.

### 2.8. Estimation of Insulin Secretion and Content

To determine insulin secretory response and content, MIN6 cells were incubated in the presence of KRB buffer and tolbutamide after which the media were collected. The supernatant was used to measure the secretion of insulin whereas the remaining cell pellets were used to analyse insulin content. Standards and samples (10 μL) were transferred onto a 96-well plate in duplicate followed by the incubation step as described in the manufacturer’s protocol (Mouse Insulin ELISA, Cat No. 10-1247-01—Mercodia, Diagenics, Milton Keynes, UK). The absorbance was measured at 450 nm using a microplate reader.

### 2.9. SNAP-25 Protein Expression

The expression of SNAP-25 protein was quantified by immunoblotting. Protein (10 μg) was loaded onto gels and following transfer, membranes were incubated with rabbit-anti-rat SNAP-25 antibody (Cat No. ab5666—Abcam, Abcam, Cambridge, UK) (1 μg/mL). Goat anti-rabbit conjugated to horseradish peroxidase (Cat No. ab205718—Abcam, Abcam, Cambridge, UK) at a dilution of 1:2000 was used as the secondary antibody. Blots were probed with goat polyclonal anti β-actin (Cat No. ab8229—Abcam, Abcam, Cambridge, UK) at 1:500 dilution as a loading control. The membrane was visualised using electrochemiluminescence Western blotting detection reagent, and analysed by scanning densitometry using Azure Biosystems 600 imaging system (Azure Biosystem, Dublin, CA, USA). Blots were quantified using imageJ (NIH, Bethesda, MD, USA). 

### 2.10. Cell Culture with Transferrin Treatments

MIN6 cell ferritin protein content was determined using a commercial ELISA kit according to the manufacturer’s instructions following treatment of cells with human transferrin (Sigma Aldrich, Poole, UK) at 0.005, 0.05, 0.5, 2, and 5 g/L for 24 h in media supplemented with tolbutamide. Iron and glucose were added as described previously in Section 2.4.

### 2.11. Statistical Analysis

The data are presented as the mean ± SEM and the differences between samples were analysed via one-way ANOVA followed by Tukey’s multiple comparisons post hoc test (PRISM software package, Version 8, GraphPad Software Inc., San Diego, CA, USA). Results were considered significantly different if *p* < 0.05.

## 3. Results

### 3.1. Assessment of Total Cellular Iron Content upon Exposure to Transferrin

**Figure 1:**
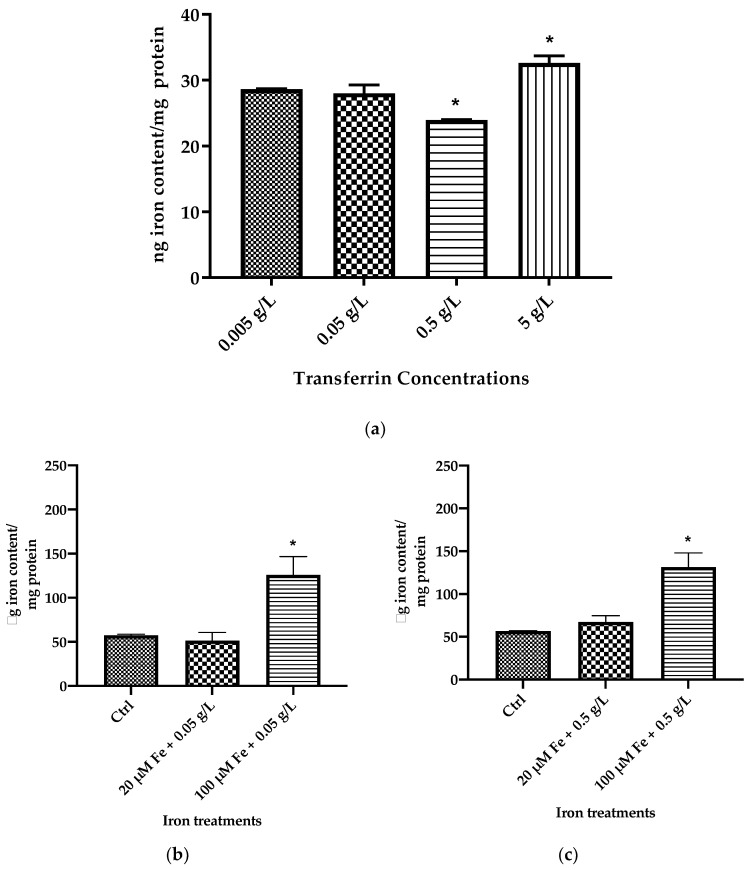

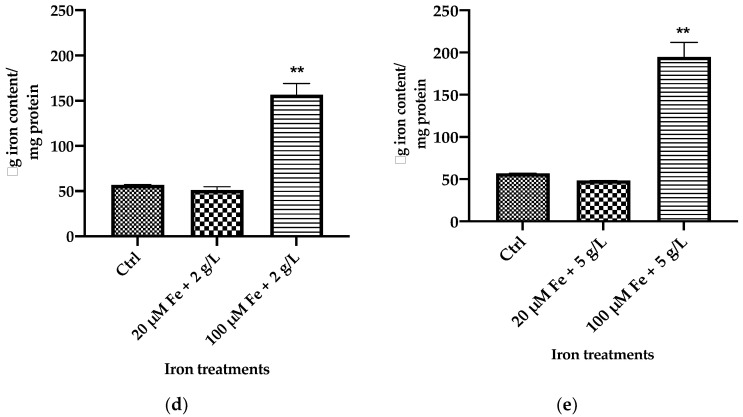
Effect of transferrin treatments alone or in combination with iron on MIN6 cell intracellular iron content. Data are presented as follows: (**a**) Dose-response effects of a range of transferrin concentrations (0.005 g/L, 0.05 g/L, 0.5 g/L and 5 g/L) loaded into MIN6 cells for 24 h. The data represent mean ± SEM, *n* = 3. * *p* < 0.018. (0.005. g/L vs. 0.5 g/L), * *p* < 0.033 (0.005 g/L vs. 5 g/L). (**b**) Effects of 0.05 g/L transferrin loaded into MIN6 cells in combination with 20 μM or 100 μM for 24 h. (**c**) Effects of 0.5 g/L transferrin loaded into MIN6 cells in combination with 20 μM or 100 μM for 24 h. (**d**)Effects of 2 g/L transferrin loaded into MIN6 cells in combination with 20 μM or 100 μM for 24 h. (**e**) Effects of 5 g/L transferrin loaded into MIN6 cells in combination with 20 μM or 100 μM for 24 h. The data (**b**–**e**) represent mean ± SEM, *n* = 3. Significances are as follows; ^*^ *p* < 0.048 (0.05 g/L),^*^ *p* < 0.0138 (0.5 g/L), ^**^ *p* < 0.0036 (2 g/L), ^**^ *p* < 0.0031 (5 g/L).

MIN6 cells were exposed to various concentrations (0.005, 0.05, 0.5, 2, and 5 g/L) of transferrin. Figure 1a shows that the highest total iron content was obtained from 5 g/L (32.42 ± 0.78 ng/mg). This was followed by 0.005 g/L (28.36 ± 0.05 ng/mg protein) and 0.05 g/L (27.5 ± 0.9 ng/mg protein) as the second and the third highest total iron content, respectively. The 0.5 g/L sample exhibited the lowest total cellular iron content (24.10 ± 0.05 ng/mg protein).

Figure 1b–e demonstrates a range of concentrations of transferrin with the addition of two selected iron concentrations (20 μM; control and 100 μM; high iron) on total cellular iron content. The data show that addition of 20 μM iron did not result in increasing iron content at most transferrin concentrations. However, 100 μM iron demonstrated higher total iron content throughout all conditions. Figure 1e represents the highest transferrin concentration (5 g/L) with the addition of 100 μM iron and leads to a significant increase (70.92 ± 17.21%) in iron content compared to 20 μM iron (*p* < 0.05). 

**Figure 2:**
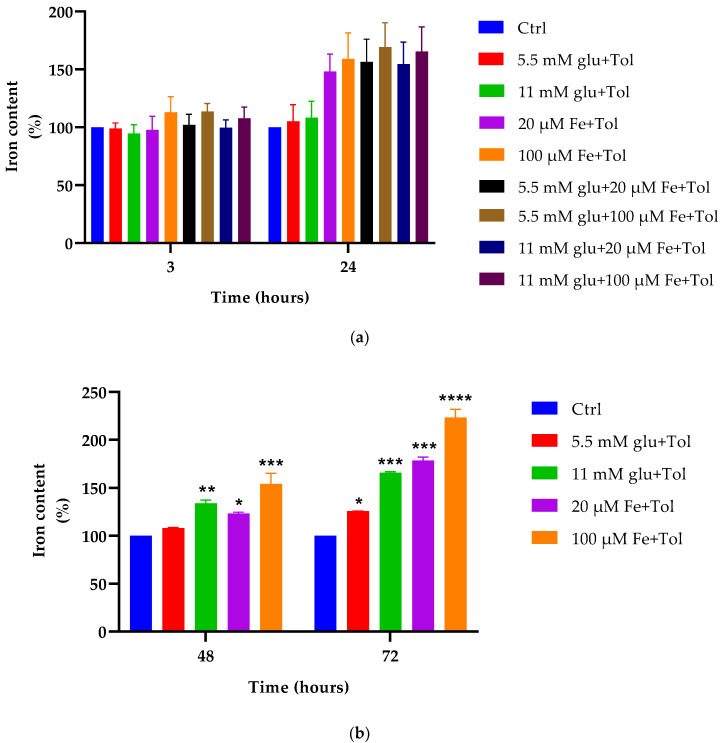
Effect varying of iron and glucose concentrations on intracellular iron content. To quantify the intracellular accumulation of iron, MIN6 cells were exposed to iron (20 μM and 100 μM) and/or glucose (5.5 mM and 11 mM) with the addition of tolbutamide as a secretagogue. Cells were incubated in a time course at 3 and 24 h (**a**), 48 and 72 h (**b**). End points measurements were determined using FerroZine™-based colorimetric assay. The data represent mean ± SEM, n = 4. Significance compared to control are as follows ** *p* < 0.004 (11 mM Glu (C2)—48 h), * *p* < 0.017 (C3—48 h), *** *p* < 0.0004 (C4—48 h); * *p* < 0.014 (5.5 mM Glu (C1)—72 h), *** *p* < 0.001 (C2—72 h), *** *p* < 0.0003 (C3—72 h), **** *p* < 0.0001 (C4—72 h).

Iron content levels were progressively decreased concomitant with decreasing transferrin concentrations. Transferrin concentration of 2 g/L showed the second highest total iron content (63.84 ± 12.52%) followed by 0.5 g/L (57.13 ± 16.70%) and 0.05 g/L (55.02 ± 20.89%), respectively. The addition of increasing transferrin concentrations to MIN6 cells thus results in elevated total cellular iron content levels. Interestingly, identical effects were observed at 48 and 72 h upon quantifying total iron content levels in the absence of transferrin (Figure 2b), although total iron content increased significantly in the presence of transferrin, particularly at 100 μM iron (Figure 1e). The reason that total iron content increased in the presence of transferrin might be due to the presence of preexisting iron-bound transferrin along with the treatment iron concentrations (20 μM or 100 μM). Thus, it is observed that the range of iron concentrations MIN6 cells were exposed to have no significant difference in cellular iron content compared to cells in the absence of transferrin.

### 3.2. Total Iron Content in the Absence of Transferrin

MIN6 cells were incubated in the presence of 20 μM (control) and 100 μM high iron or physiological and supraphysiological glucose (5.5 mM and 11 mM, respectively), or combinations of iron and glucose, all with the addition of tolbutamide for 3, 24, 48, and 72 h. As shown in Figure 2, iron or glucose or combinations thereof demonstrated no change on intracellular iron content compared to control (3 h). At 24 h, iron and glucose concentrations exhibited a trend towards higher iron content compared to control, however these effects were not statistically significant (*p* > 0.05). The highest iron content was exhibited upon treatment with 100 μM iron and 5.5 mM glucose and resulted in a 41 ± 17.1% increase compared to control (24 h). 

Furthermore, iron content was significantly increased at 48 and 72 h at 100 μM iron and 11 mM glucose concentrations compared to control (*p* < 0.05). However, 5.5 mM glucose did not demonstrate a significant effect (*p* > 0.05) compared to control. The 100 μM concentration exhibited a 34.44 ± 4.65% increase in iron content compared to control (48 h incubation). Similar to 48 h incubation, 100 μM iron and 11 mM glucose concentrations alone demonstrated significant changes, with 100 μM iron exhibiting a 2-fold increase compared to control (76.32 ± 2.66 nmol/mg protein vs. 34.80 ± 0.38 nmol/mg protein and 55.13 nmol/mg protein ± 0.93 vs. 34.80 ± 0.38 nmol/mg protein, respectively) (72 h). 

These data suggest that excess iron in the presence of glucose may exert effects on MIN6 cell iron metabolism after 24 h. 

### 3.3. Cellular Ferritin Content

**Figure 3:**
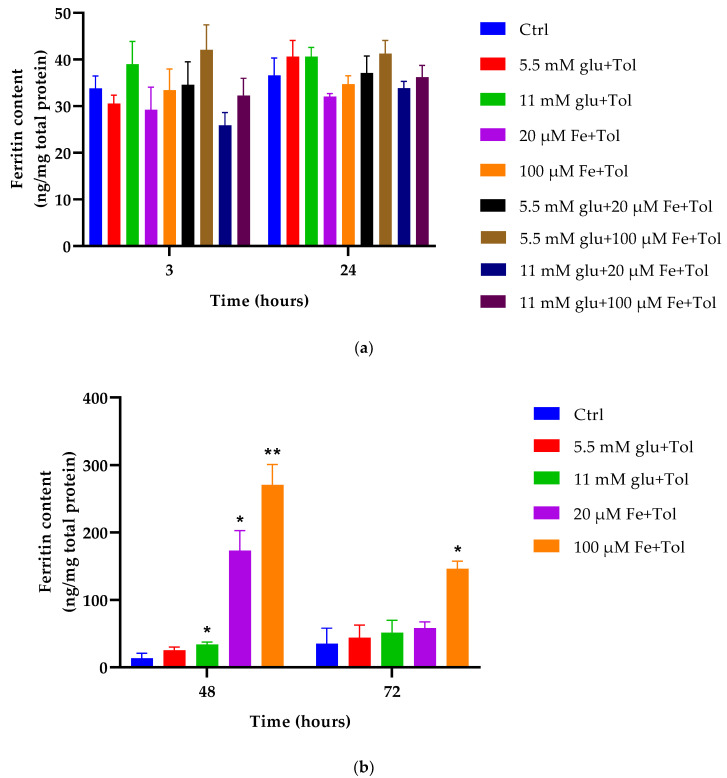
The effect of iron (20 μM and 100 μM), glucose (5.5 mM and 11 mM), and combinations of iron and glucose, all with the addition of tolbutamide as a secretagogue on MIN6 cells on ferritin content. This assessment was performed at four different time points [3 and 24 h (**a**), and 48 and 72 h (**b**)]. The data represent mean ± SEM, *n* = 4. Significance compared to control is as follows * *p* < 0.028 (11 mM Glu (C2)—48 h), * *p* < 0.023 (20 μM Fe (C3)—48 h), ** *p* < 0.006 (100 μM Fe (C4)—48 h); * *p* < 0.025 (C4—72 h).

As shown in Figure 3, iron and glucose demonstrated no effect on MIN6 cell ferritin content at 3 h incubation. In addition, conditions containing iron and glucose in combination also exhibited no change in ferritin content. Glucose treatment appeared to slightly increase ferritin content and iron slightly reduced it at 3 h. Similar effects remained observable at 24 h at which iron, glucose, and combinations of iron and glucose demonstrated no short-term effects on iron storage. 

All iron (20 μM and 100 μM) and glucose (5.5 and 11 mM) treatments led to significant increases in ferritin content at 48 h. High iron exhibited 15-fold higher ferritin content than its control (271 ± 30.40 ng/mg protein vs. 17.81 ± 7.80 ng/mg protein), which was followed by 20 μM iron with a 5-fold increase (173.02 ± 29.91 ng/mg protein vs. 17.81 ± 7.81 ng/mg protein). Moreover, 11 mM glucose demonstrated ferritin content almost twice as high as control (47.72 ± 3.50 %). In contrast, 5.5 mM glucose showed no effect on ferritin content compared to control at 72 h incubation. At high iron, ferritin content was significantly increased at levels four times higher (76.13 ± 11.15%) than its control (*p* < 0.05). Thus, MIN6 cells exposed to high iron concentration (100 μM) result in high ferritin content at both 48 h and 72 h.

### 3.4. Estimation of Lipid Peroxidation 

**Figure 4:**
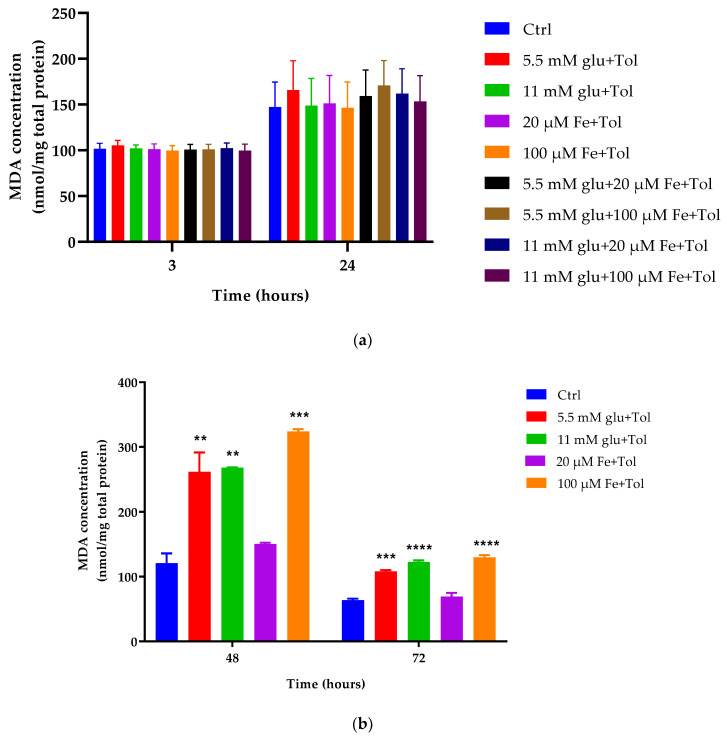
Determination of lipid peroxidation marker (MDA) performed by TBARS assay (**a**) following 3 h and 24 h incubation with test conditions; iron (20 μM and 100 μM) and/or glucose (5.5 mM and 11 mM), and combinations of iron and glucose and (**b**) following 48 h and 72 h incubation with these test conditions (b). High iron and glucose (100 μM and 11 mM respectively) in presence of tolbutamide as a secretagogue increased the concentration of MDA compared to control both at 48 and 72 h time points. The data represent mean ± SEM; *n* = 4. ** *p* < 0.0012 (5.5 mM glu—48 h), ** *p* < 0.001 (11 mM glu—48 h), *** *p* < 0.0002 (100 μM Fe—48 h), *** *p* < 0.0003 (5.5 mM glu—72 h), **** *p* < 0.0001 (11 mM glu and 100 μM Fe—72 h).

Quantification of cellular lipid peroxidation is crucial to providing information on the effects of ROS-mediated oxidative stress. As shown in Figure 4, iron (20 μM and 100 μM) and glucose (5.5 mM and 11 mM) demonstrated no effects on MDA levels both at 3 and 24 h incubation. Although slight changes were observed at glucose concentrations (5.5 mM and 11 mM) alone and conditions containing iron and glucose combinations, MDA levels were increased not significantly compared to control (*p* > 0.05) (24 h). In contrast, MDA levels were significantly increased at high iron (100 μM) and glucose concentrations (5.5 mM and 11 mM) at both 48 and 72 h incubation (*p* < 0.05).

High iron and high glucose concentrations exhibited more than 50% higher MDA levels compared to its control with high iron demonstrating the highest MDA level, 62.73% higher than its control (3323.87 ± 0.82 nmol/mg protein vs. 120.82 ± 2.47 nmol/mg protein). The effect remained at 72 h incubation at which high iron and high glucose exhibited significantly increased levels of MDA compared to control (*p* < 0.05). Consistent with the result at 48 h, high iron resulted in the highest MDA level with a 51% increase compared to control (129 ± 0.81 nmol/mg protein vs. 63.7 ± 0.52 nmol/mg protein). 

### 3.5. Assessment of Cellular Viability

**Figure 5:**
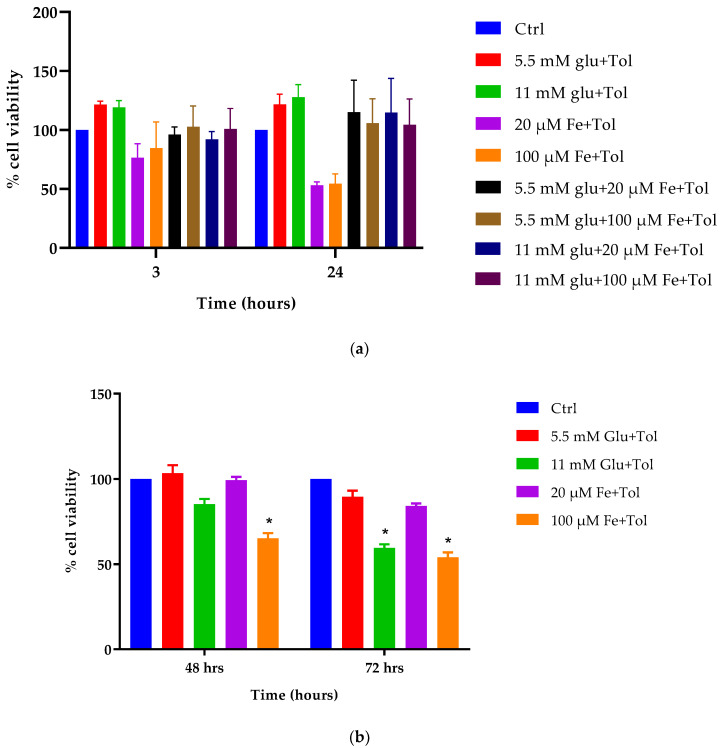
The effect of iron and glucose on MIN6 cell viability. Cytotoxicity assessment was performed by PrestoBlue^®^ assay at four incubation times [(3 and 24 h (**a**), 48 and 72 h (**b**)]. MIN6 cells were exposed to various concentrations of iron (20 μM and 100 μM) and/or glucose (5.5 mM and 11 mM), and combinations of iron and glucose with the addition of tolbutamide as a secretagogue. The data represent mean ± SEM, *n* = 4. * *p* < 0.02 represents significance compared to control.

The results shown in Figure 5 demonstrate the effects of various concentrations of iron, glucose, and combinations of iron and glucose with the addition of tolbutamide on MIN6 cell viability at 3, 24, 48, and 72 h. Exposure to both 5.5 mM and 11 mM glucose concentrations exhibited significantly increased cell viability, 21.63% and 19.21% higher than the control, respectively (3 h incubation). Although iron concentrations demonstrated a decreased percentage of cell viability, the levels were not significant (*p* > 0.05). At 24 h incubation, similar effects were observed with both iron and glucose concentrations; glucose concentrations increased cell viability, but iron concentrations resulted in a decrease. However, glucose concentrations exerted a non-significant effect compared to control, whereas both 20 μM and high 100 μM iron demonstrated significant reductions (47.15% and 46.27%, respectively).

At 48 h incubation, glucose concentrations demonstrated slight changes, in which 5.5 mM glucose demonstrated a non-significant increase in cell viability (*p* > 0.05), but high 11 mM glucose resulted in the opposite effect. In contrast, high iron (100 μM) concentration significantly reduced the percentage of cell viability, with a 35% reduction compared to control. Similar effects were also observed at 72 h at which high iron exhibited a 46% reduction of cell viability, followed by high glucose with 40.54% reduction in cell viability. These results are generally consistent with data obtained in previous analyses, suggesting high iron and high glucose may lead to impairments of cellular function.

### 3.6. Assessment of Insulin Content

**Figure 6:**
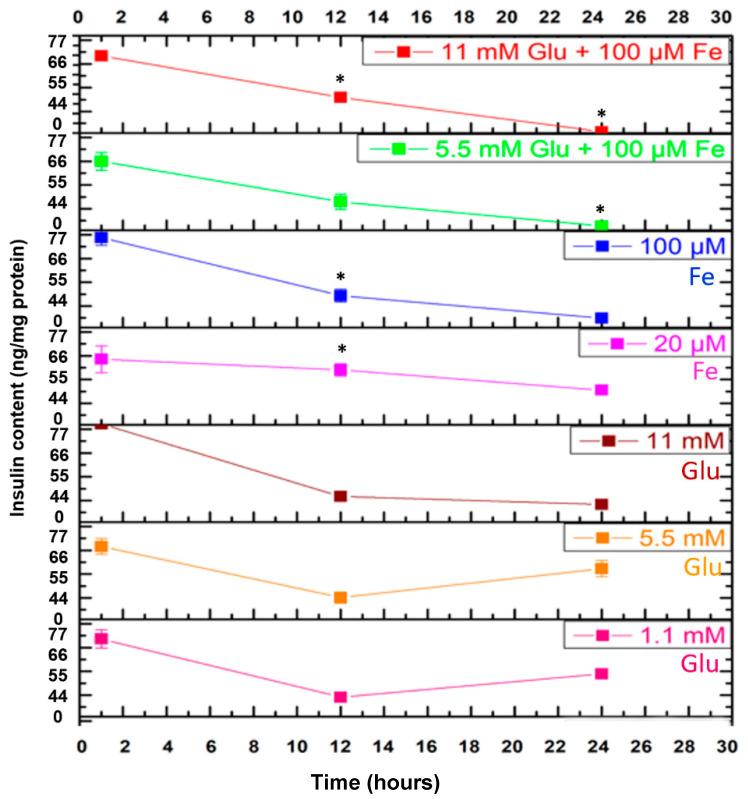
The effect of iron and glucose on intracellular insulin content. Measurements were performed using insulin ELISA in cultured cells. MIN6 cells were exposed to various concentrations of iron, glucose, and combinations of iron and glucose with the addition of tolbutamide at designated incubation times. The data represent mean ± SEM, *n* = 3. * *p* < 0.0193 (20 μM Fe (C3)—12 h), * *p* < 0.022 (100 μM Fe (C4)—12 h), * *p* < 0.0148 (11 mM Glu + 100 μM Fe (C8)—12 h); * *p* < 0.0229 (5.5 mM Glu + 100 uM Fe (C6)—24 h), * *p* < 0.011 (11 mM Glu + 100 μM Fe (C8)—24 h).

Insulin content was quantified to determine the balance between insulin synthesis and secretion. As shown in Figure 6, iron and glucose slightly altered the level of intracellular insulin content in which the values were increased, although non-significantly compared to control following a 1 h incubation (*p* > 0.05). In addition, the combinations of iron and glucose concentrations demonstrated no change in insulin content. In contrast, following 12 h incubation, 100 μM iron (53.01 μg/mg protein vs. 38.13 μg/mg protein) exhibited a significant increase in insulin content compared to control (Figure 6). Glucose treatment (11 mM) demonstrated higher insulin content but was not statistically significant (*p* > 0.05).

At 24 h incubation, both 20 μM and 100 μM iron led to a reduction in insulin content that was not statistically significant (20 μM Fe: 12.27%; 100 μM Fe: 10.22%), whereas 5.5 mM glucose led to a slight increase that was not statistically significant (5.5 mM Glu: 4%). Iron and glucose had no influence on insulin content at 1 h but began to exert significant effects at 12 h following treatment incubation. At 24 h, iron and glucose demonstrated contrasting effects; iron treatment showed a trend towards reduced insulin content whereas glucose treatments increased it.

### 3.7. Assessment of Insulin Secretion

**Figure 7:**
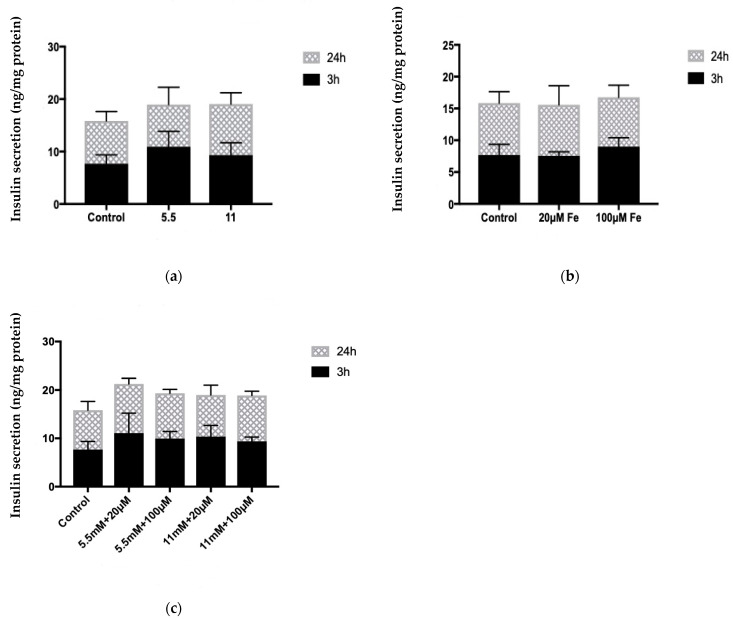

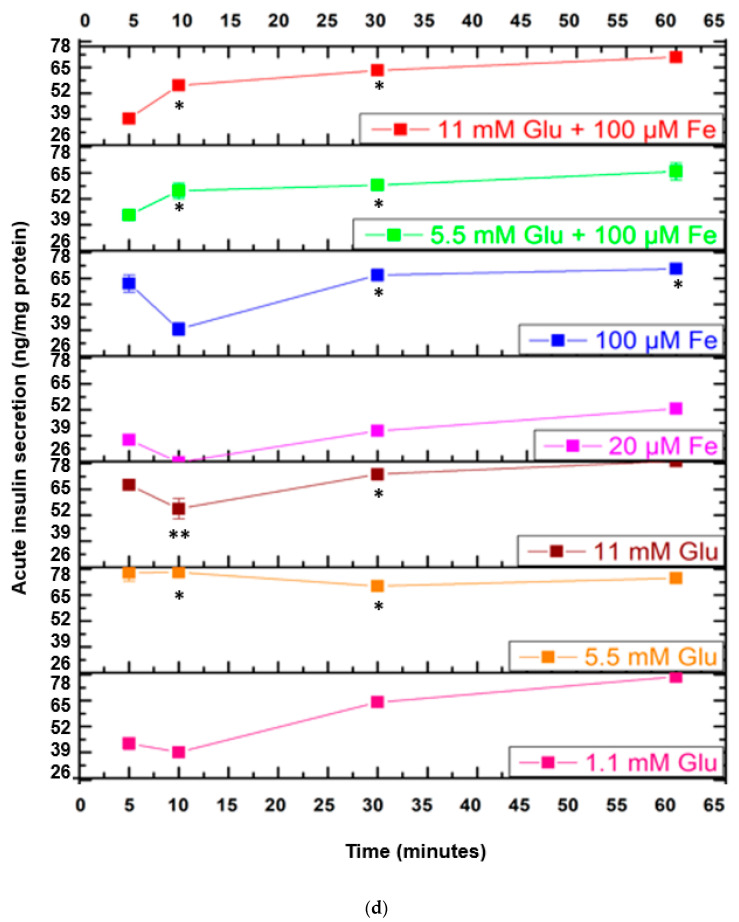

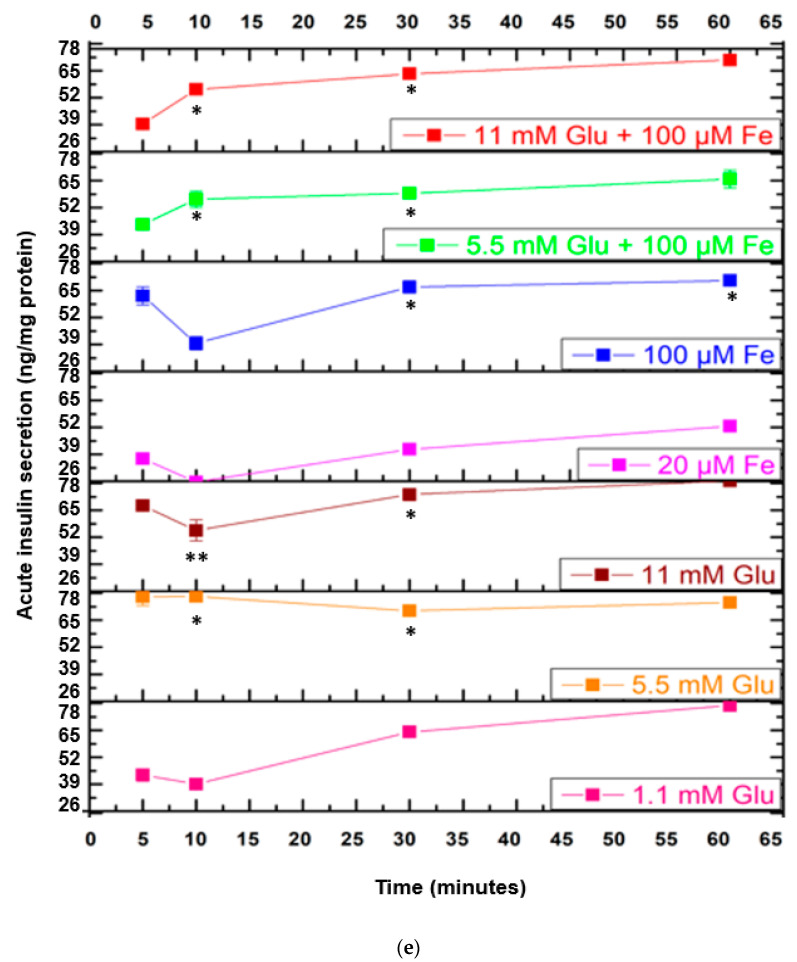
The effect of treatment with varying concentrations of iron, glucose, and combinations of iron and glucose with the addition of tolbutamide on MIN6 cells insulin secretion. (**a**) The effect of treatment with 5.5 mM and 11 mM of glucose with the addition of tolbutamide on MIN6 cells insulin secretion following 3 h and 24 h incubation. The data represent mean ± SEM, *n* = 3. (**b**) The effect of treatment with 20 μM and 100 μM iron with the addition of tolbutamide on MIN6 cells insulin secretion following 3 h and 24 h incubation. The data represent mean ± SEM, *n* = 3. (**c**) The effect of treatment with 5.5 mM and 11 mM of glucose and/or 20 μM and 100 μM iron and combinations of iron and glucose with the addition of tolbutamide on MIN6 cells insulin secretion following 3 h and 24 h incubation. The data represent mean ± SEM, *n* = 3. (**d**) The effect of treatment incubation with varying concentrations of iron, glucose, and combinations of iron and glucose with the addition of tolbutamide on MIN6 cells insulin secretion acutely (5, 10, 30, 60 min). The data represent mean ± SEM, *n* = 3. * *p* < 0.0036 (5.5 mM Glu (C1)—10 min), ** *p* < 0.037 (11 mM Glu (C2)—10 min), ** *p* < 0.005 (20 μM Fe (C3)—10 min), * *p* < 0.026 (5.5 mM Glu + 20 μM Fe (C6)—10 min), * *p* < 0.01 (11 mM Glu + 100 μM Fe (C8)—10 min); * *p* < 0.05 (C1—30 min), * *p* < 0.033 (C2—30 min), * *p* < 0.016 (C3—30 min), * *p* < 0.026 (C6—30 min), * *p* < 0.01 (C8—30 min); * *p* < 0.013 (C3—60 min). (**e**) The effect of treatment with varying concentrations of iron, glucose, and combinations of iron and glucose with the addition of tolbutamide on MIN6 cells insulin secretion following 24 h (5, 10, 30, 60 min). The data represent mean ± SEM, *n* = 3. * *p* < 0.049 [11 mM Glu (C2)—24 h (5 min)], * *p* < 0.022 [20 μM Fe (C3)—24 h (5 min)], ** *p* < 0.004 [100 μM Fe (C4)—24 h (5 min)], * *p* < 0.013 [5.5 mM Glu + 100 μM Fe—24 h (C6)(5 min)], ** *p* < 0.003 [11 mM Glu + 100 μM Fe—24 h (C8)(5 min)]; * *p* < 0.034 [C2—24 h(10 min)], * *p* < 0.022 [C3—24 h(10 min)], * *p* < 0.02 (C4—24 h(10 min)], ** *p* < 0.005 [C6—24 h(10 min)], * *p* < 0.027 [C8—24 h(10 min)]; * *p* < 0.015 [C2—24 h(30 min)], ** *p* < 0.004 [C3—24 h(30 min)], * *p* < 0.016 [C4—24 h(30 min)], ** *p* < 0.005 [C6—24 h(30 min)], * *p* < 0.018 [C8—24 h(30 min)]; * *p* < 0.044 [C2—24 h(60 min)], * *p* < 0.034 (C3, C4, C6—24 h(60 min)].

The data in Figure 7 showed insulin secretion levels upon exposure to various conditions of iron, glucose, and combinations of iron and glucose at 5, 10, 30, and 60 min. 

The results show insulin secretion levels following treatment with glucose compared to control (Figure 7a), iron against control (Figure 7b), combinations of iron and glucose against control (Figure 7c), and measurements of iron, glucose, and combinations of iron and glucose at 5, 10, 30, and 60 min (acute) (Figure 7d), which was also applied at 24 h incubation (long term) (Figure 7e). 

As shown in Figure 7a–c, both 5.5 mM and 11 mM glucose concentrations (5.5 mM: 10.50 μg/mg protein vs. 9.02 μg/mg protein; 11 mM: 9.73 μg/mg protein vs. 9.02 μg/mg protein) slightly increased insulin secretion both at 3 and 24 h (Figure 7a). The 20 μM concentration of iron had no effect on insulin secretion (8.73 μg/mg protein vs. 8.71 μg/mg protein), while 100 μM iron showed a trend towards increased insulin secretion (9.26 μg/mg protein vs. 8.71 μg/mg protein) compared to control (3 h) (Figure 7b). This was also observed at 24 h incubation. Furthermore, insulin secretion was slightly increased in all conditions containing iron and glucose combinations compared to control. However, the treatment containing 20 μM iron and 5.5 mM glucose combinations exhibited the highest insulin secretion at both 3 and 24 h incubation (10.2 µg/mg protein vs. 8.27 µg/mg protein, 33 µg/mg protein vs. 17.70 µg/mg protein, respectively) (Figure 7c). 

Short-term incubations were conducted on MIN6 cells where changes occurred under all conditions at several incubations. At 5 min incubation, 100 μM iron and 11 mM glucose exhibited higher insulin secretion with 33% and 26.2% compared to control, respectively.

In addition, the combinations of iron and glucose demonstrated a trend towards reduced insulin secretion compared to control (*p* > 0.05). However, 20 μM iron reduced insulin secretion by 33.31%, whereas 100 μM iron resulted in no change compared to control (10 min incubation). Insulin secretion (5.5 mM: 9.59% and 11 mM: 14%) showed a trend towards significance following treatment with both glucose concentrations (*p* > 0.05). At 30 min incubation, 100 μM iron and 11 mM glucose increased insulin secretion non-significantly (*p* > 0.05). At 30 min incubation, 100 μM iron and 11 mM glucose increased insulin secretion non-significantly (*p* > 0.05). In contrast, 20 μM iron reduced insulin secretion by 13.34%, but secretion was increased (18.82%) at 100 μM iron and with 11 mM glucose which had the highest insulin secretion (23.11% compared to control) (60 min incubation) (Figure 7d).

Acute changes at 5, 10, 30, and 60 min were investigated and insulin secretion was measured (Figure 7e). Here, 100 μM iron alone and 11 mM glucose alone demonstrated significant reductions in levels of insulin secretion (63.53%, 56.08%, 74.53, 47.21% and 31.71%, 35.13%, 34.50%, 43.42%, respectively) across various time courses compared to control (*p* > 0.05). This was also shown by conditions containing iron and glucose combinations.

### 3.8. Expression of SNAP-25–Mediated Insulin Exocytosis

**Figure 8:**
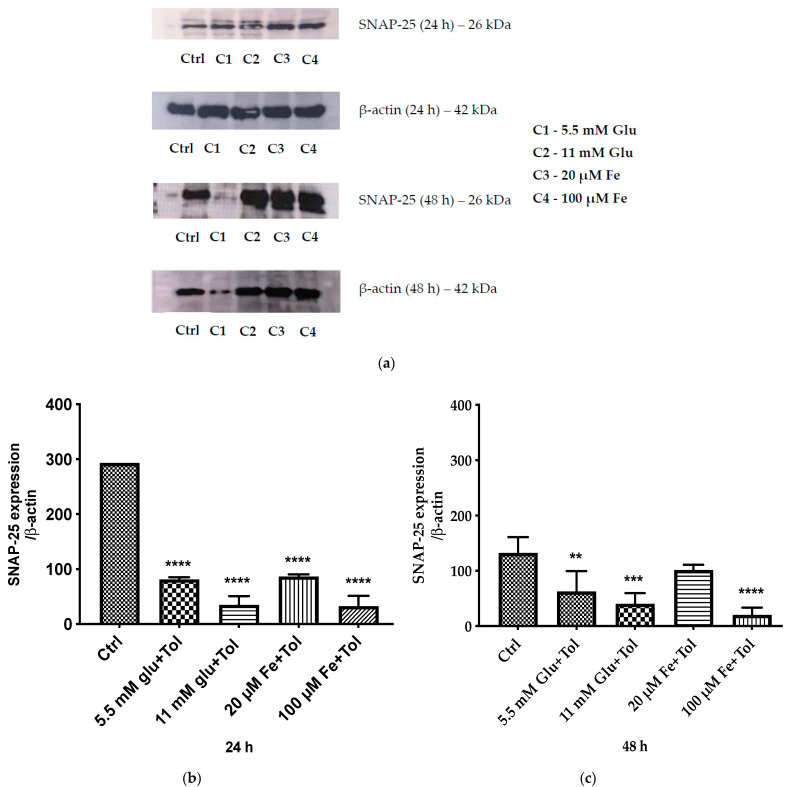
SNAP-25 protein expressions in pancreatic β-cell (MIN6) within two distinct timelines, 24 and 48 h, in the presence of iron (20 μM and 100 μM) and glucose (5.5 mM and 11 mM) with the addition of tolbutamide as a secretagogue. Protein expression was identified using Western blotting. (**a**) Distinctive bands of SNAP-25 (24 and 48 h) and β-actin (24 and 48 h). Gel loading was as follows (from top left): lane 1—ctrl, lane 2—normal glucose (5.5 mM) (C1), lane 3—high glucose (11 mM) (C2), lane 4—normal iron (20 μM) (C3), lane 5—high iron (100 μM) (C4); (from middle right): lane 1—ctrl, lane 2—C1, lane 3—C2, lane 4—C3, lane 5—C4. (**b**) Expression of SNAP-25 at 24 h normalised against β-actin. (**c**) Expression of SNAP-25 at 48 h normalised against β-actin. The data represent mean ± SEM; *n* = 4. **** *p* < 0.0001 (24 h), ** *p* < 0.0061 (48 h), *** *p* < 0.0005, **** *p* < 0.0001 (48 h).

MIN6 cells were exposed to pre-determined concentrations of iron (20 μM and 100 μM) and glucose (5.5 mM and 11 mM) for 24 and 48 h. Figure 8a–c depicts the measurements of SNAP-25 protein expression using immunoblotting. The high iron and glucose concentration decreased the SNAP-25 expression levels at both time points, compared to that in the control. The high iron concentration produced the lowest expression of this particular protein in comparison to that under all the other conditions at both 24 and 48 h.

A significant reduction in MIN6 β-cell SNAP-25 protein expression was evident at 24 h upon exposure to 100 µM iron (65 ± 13.09 vs. 293 ± 0.42) and 11 mM glucose (34.71 ± 11.30 vs. 293 ± 0.42). A similar expression of SNAP-25 was also observed after 48 h of incubation, at which high iron and high glucose concentrations induced significant reductions compared to that in the control (48.40 ± 6.52 vs. 132.45 ± 14.33 and 43.13 ± 9.52 vs. 132.45 ± 14.3, respectively). Moreover, 20 μM iron concentration did not significantly decrease SNAP-25 expression levels compared to that in the control (62.62 ± 4.70 vs. 132.45 ± 14.33) after 48 h of incubation. However, after 24 h of incubation, it decreased the SNAP-25 levels significantly compared to that in the control (86.61 ± 2.71 vs. 293 ± 0.42). These results suggest that 100 µM iron and 11 mM glucose concentrations significantly reduce the levels of SNAP-25 in MIN6 cells even after 24 h.

## 4. Discussion

Diabetes mellitus is a complex disease with multiple complications affecting people of different ages and sex [21]. Iron is an important nutrient that has been identified as one of the factors contributing to the progression of T2DM, although the mechanisms are not well clarified. Iron participates in many biological processes in the human body, including playing an essential role in the production of ATP and metabolic processes such as DNA repair and replication, and regulation of gene expression [22]. Higher levels of intracellular iron can be involved in the regulation of cell proliferation by regulating transcription factors, controlling cell cycle progression and apoptosis [23]. 

### 4.1. Exposure of MIN6 β-Cells to High Iron Levels Results in Significantly Elevated Cellular Iron Accumulation 

Iron metabolism, which involves its uptake, distribution, storage, and secretion, has been well established. Ferric iron is normally bound to apotransferrin forming diferric-Tf, with the purpose of distributing iron throughout the body. The experiments summarised in Figure 1 demonstrated that although the addition of transferrin bound to iron resulted in significant levels of total iron content intracellularly (*p* < 0.05), the same effects remained in its absence (Figure 2), demonstrating that high iron (100 μM) led to overall higher total iron content compared to all other cell treatments. Once iron enters into cells, it can be transported into mitochondria or stored within the cytoplasm as ferritin. Ferritin is considered a major store of intracellular iron, with storage being a key component of iron metabolism. This protein is fundamental in controlling the amount of iron available to the body. It has the ability to store and release iron in a controlled fashion, which helps to prevent iron disorders like iron overload, anaemia, and other iron-related diseases. Serum ferritin is a crucial measurement as this protein is commonly used as an indicator of tissue iron status. The results illustrated in Figure 3 show ferritin content increased in an iron concentration-dependent manner, when incubated at 48 h (fifteen times higher) and 72 h (four times higher) with excess iron. These data correspond to total iron content (Figure 2) performed by colorimetric FerroZine™ assay, where similar results were obtained.

Ford and Cogswell elucidated that an increase in ferritin has been positively correlated with the risk of developing T2DM [24]. This study is also supported by Aregbesola and colleagues, who suggested that ferritin levels above normal were related to an increased risk of diabetes mellitus [25]. The potential reason for increased ferritin in the β-cells may be due to the fact that ferritin exhibits antioxidant properties as it acts as a protective molecule against iron toxicity by sequestering excess free iron [26]. This is particularly relevant as β-cells are more sensitive to reactive oxygen radicals due to lack of substantial intracellular antioxidant enzyme defenses. Iron can be released from ferritin, converting Fe^3+^ into the more reactive Fe^2+^ form by the action of a reducing agent, and the release of reactive free iron may occur at a faster rate than what can be countered by the endogenous antioxidants [27]. Under this condition, elevated levels of free iron are released from ferritin. Interestingly, this phenomenon has also been observed in pre-diabetes, concomitant with low transferrin saturation levels [28]. The link between increased serum ferritin levels and the risk of diabetes mellitus remains to be fully understood. However, oxidative stress initiated by the release of excess free iron that acts as a catalyst for ROS generation may be a putative mechanism.

### 4.2. Excess Iron in MIN6 β-Cells Results in ROS Generation, Lipid Peroxidation and Cytotoxic Effects 

In the present study, excess iron-generated ROS was shown to increase MDA (measured by TBARS) contributing to apoptosis initiation. Figure 4 showed that excess iron demonstrated higher MDA levels, which started at 48 h incubation likely as a result of ROS-mediated cellular lipid peroxidative effects. Moreover, another major aldehyde product of this structure is 4-hydroxy-2-nonenal (HNE), characterised as a major toxic product and considered as weakly mutagenic [29]. It can be seen that there is a profound increase in MDA levels in the presence of excess iron, which suggests that there is increased production of free radicals [30]. These free radicals promote lipid peroxidation, resulting in the chain reaction that ultimately leads to damaging various molecules, eventually leading to cellular dysfunction [30]. 

As mentioned previously, iron has a dual role—it is essential in both cellular metabolism and aerobic respiration. However, the involvement of this metal leads to the formation of free radicals that can cause cellular toxicity and oxidative damage on the cellular components. The effect of excess iron and glucose on the viability of the cells was assessed (Figure 5). In line with the previous results, toxicity caused by excess iron exhibited a significant reduction in cell viability compared to control cells, both at 48 and 72 h (*p* < 0.05). This toxicity results from the production of ROS, causing β-cell dysfunction via decreased insulin gene expression [31]. If β-cells were exposed to these concentrations for a prolonged and repeated period, they may undergo exhaustion, promoting the inhibition of insulin secretion, and leading to irreversible damage. Furthermore, due to tight regulation of iron in mitochondria, it is susceptible to the generation of reactive free radicals even in conditions of minor elevations in iron levels. ROS mediates apoptosis of pancreatic islets, decreasing their capacity to secrete optimal levels of insulin [32].

### 4.3. Excess Iron in MIN6 β-Cells Results in Dysfunctions in Insulin Secretory Machinery

Iron is known to be involved in the insulin secretory mechanism. Elevated iron levels can generate free radicals and formation of ROS. Figure 7 shows that excess iron progressively decreased secretion of insulin after a 24 h incubation. This could be caused by impairment of insulin secretion because of an elevated and continuous exposure of intracellular ROS [33]. Similar effects are also observed in measurements of insulin content (Figure 6). Impairment of insulin secretion and insulin resistance can lead to hyperglycaemia, which is a known feature of T2DM. A study performed by Shaaban and colleagues (2016) concluded that excess iron negatively impacted insulin action in healthy people [34]. Furthermore, a considerable body of evidence indicates that excess iron increases the risk for insulin resistance in diabetes mellitus, as well as other diseases such as cardiovascular diseases both in nondiabetic and diabetic individuals [34]. 

The ultimate effect of chronic impairment of insulin secretion due to excess iron and glucose is oxidative stress in the mitochondria, and finally apoptosis [35]. When β-cells sense the presence of glucose, the cytosolic levels of Ca^2+^ increase owing to molecular processes. This increase stimulates and activates the SNARE machinery, which mediates insulin granule fusion with the plasma membrane [36]. It has been observed that SNARE core complexes, key proteins involved in insulin exocytosis, are likely to be sensitive towards oxidative stress [37]. It has also been shown previously that hydrogen peroxide as an endogenously generated ROS inhibits insulin secretion in isolated pancreatic islets [38]. SNAP-25 has been shown to be most sensitive to modification by ROS, possibly due to its distinctive structure, which is unable to anchor into the membrane due to the absence of a transmembrane segment [39]. Figure 8 shows that SNAP-25 expression diminishes in MIN6 cells in the presence of high iron and glucose concentrations for 24 and 48 h, compared to that in the control. A significant shift is apparent even at 24 h. These data are in accordance with earlier data collected in this study, which indicate that high iron and glucose concentrations significantly decrease insulin secretion (24 h) (*p* < 0.05) (Figure 7e). The alteration of this protein may cause insulin secretion perturbations associated with the regulation of Ca^2+^ dynamics and membrane potential in the β-cells [40]. Studies by others have shown that enhancing the level of exocytotic proteins can improve the longevity of cells and reduce the occurrence of diseases such as diabetes mellitus and neurodegenerative disorders [41].

### 4.4. Conclusions

In summary, these data demonstrate that pancreatic β-cells are susceptible to excessive iron accumulation, which results in a reduction in cell viability and diminishing MIN6 β-cell insulin secretion by causing reduced expression of key exocytotic proteins. Thus, this work further supports the notion of a mechanistic role for β-cell iron overload in the development and progression of T2DM. Further experiments are required to investigate this mechanism and the role of the mitochondria under iron-induced oxidative stress. 

## Figures and Tables

**Table 1:** Iron and glucose treatment conditions.

Name of Condition	Treatment	Glucose Concentrations (mM)	Iron Concentrations (µM)
Ctrl	Basal Glu	1.1	0
C1	Glu	5.5	0
C2	Glu	11	0
C3	Fe	0	20
C4	Fe	0	100
C5	Glu + Fe	5.5	20
C6	Glu + Fe	5.5	100
C7	Glu + Fe	11	20
C8	Glu + Fe	11	100

## Supplementary Materials

The following are available online at https://www.mdpi.com/article/10.3390/cells10051141/s1, Figure S1: Growth pattern of MIN6 cells into mature pseudoislets.

## References

[1] Guariguata L., Whiting D.R., Hambleton I., Beagley J., Linnenkamp U., Shaw J.E. (2014). Global Estimates of Diabetes Prevalence for 2013 and Projections for 2035. Diabetes Res. Clin. Pract..

[2] Wild S., Roglic G., Green A., Sicree R., King H. (2004). Global Prevalence of Diabetes: Estimates for the Year 2000 and Projections for 2030. Diabetes Care.

[3] Tripathy D., Chavez A.O. (2010). Defects in Insulin Secretion and Action in the Pathogenesis of Type 2 Diabetes Mellitus. Curr. Diab. Rep..

[4] Jewell J.L., Oh E., Thurmond D.C. (2010). Exocytosis Mechanisms Underlying Insulin Release and Glucose Uptake: Conserved Roles for Munc18c and Syntaxin 4. Am. J. Physiol. Regul. Integr. Comp. Physiol..

[5] Gerber P.A., Rutter G.A. (2017). The Role of Oxidative Stress and Hypoxia in Pancreatic Beta-Cell Dysfunction in Diabetes Mellitus. Antioxid. Redox Signal..

[6] Lenzen S., Drinkgern J., Tiedge M. (1996). Low Antioxidant Enzyme Gene Expression in Pancreatic Islets Compared with Various Other Mouse Tissues. Free Radic. Biol. Med..

[7] Pizzino G., Irrera N., Cucinotta M., Pallio G., Mannino F., Arcoraci V., Squadrito F., Altavilla D., Bitto A. (2017). Oxidative Stress: Harms and Benefits for Human Health. Oxid. Med. Cell. Longev..

[8] McCord J.M. (2000). The Evolution of Free Radicals and Oxidative Stress. Am. J. Med..

[9] Ayala A., Muñoz M.F., Argüelles S. (2014). Lipid Peroxidation: Production, Metabolism, and Signaling Mechanisms of Malondialdehyde and 4-Hydroxy-2-Nonenal. Oxid. Med. Cell. Longev..

[10] Jezek P., Hlavatá L. (2005). Mitochondria in Homeostasis of Reactive Oxygen Species in Cell, Tissues, and Organism. Int. J. Biochem. Cell Biol..

[11] Ma Z.A., Zhao Z., Turk J. (2012). Mitochondrial Dysfunction and β-Cell Failure in Type 2 Diabetes Mellitus. Exp. Diabetes Res..

[12] Simcox J.A., McClain D.A. (2013). Iron and Diabetes Risk. Cell Metab..

[13] Jellinger K., Paulus W., Grundke-Iqbal I., Riederer P., Youdim M.B. (1990). Brain Iron and Ferritin in Parkinson’s and Alzheimer’s Diseases. J. Neural Transm. Park. Dis. Dement. Sect..

[14] Andrews N.C. (1999). Disorders of Iron Metabolism. N. Engl. J. Med..

[15] Fairweather-Tait S.J. (2004). Iron Nutrition in the UK: Getting the Balance Right. Proc. Nutr. Soc..

[16] Hansen J.B., Moen I.W., Mandrup-Poulsen T. (2014). Iron: The Hard Player in Diabetes Pathophysiology. Acta Physiol..

[17] Lenzen S. (2008). Oxidative Stress: The Vulnerable Beta-Cell. Biochem. Soc. Trans..

[18] Jiang R., Manson J.E., Meigs J.B., Ma J., Rifai N., Hu F.B. (2004). Body Iron Stores in Relation to Risk of Type 2 Diabetes in Apparently Healthy Women. JAMA.

[19] Johnson D., Shepherd R.M., Gill D., Gorman T., Smith D.M., Dunne M.J. (2007). Glucose-Dependent Modulation of Insulin Secretion and Intracellular Calcium Ions by GKA50, a Glucokinase Activator. Diabetes.

[20] Riemer J., Hoepken H.H., Czerwinska H., Robinson S.R., Dringen R. (2004). Colorimetric Ferrozine-Based Assay for the Quantitation of Iron in Cultured Cells. Anal. Biochem..

[21] Kautzky-Willer A., Harreiter J., Pacini G. (2016). Sex and Gender Differences in Risk, Pathophysiology and Complications of Type 2 Diabetes Mellitus. Endocr. Rev..

[22] Paul B.T., Manz D.H., Torti F.M., Torti S.V. (2017). Mitochondria and Iron: Current Questions. Expert Rev. Hematol..

[23] Evan G.I., Vousden K.H. (2001). Proliferation, Cell Cycle and Apoptosis in Cancer. Nature.

[24] Ford E.S., Cogswell M.E. (1999). Diabetes and Serum Ferritin Concentration among U.S. Adults. Diabetes Care.

[25] Aregbesola A., Voutilainen S., Virtanen J.K., Mursu J., Tuomainen T.-P. (2013). Body Iron Stores and the Risk of Type 2 Diabetes in Middle-Aged Men. Eur. J. Endocrinol..

[26] Juckett M.B., Balla J., Balla G., Jessurun J., Jacob H.S., Vercellotti G.M. (1995). Ferritin Protects Endothelial Cells from Oxidized Low Density Lipoprotein in Vitro. Am. J. Pathol..

[27] Halliwell B. (1993). The Role of Oxygen Radicals in Human Disease, with Particular Reference to the Vascular System. Haemostasis.

[28] Cheung C.-L., Cheung T.T., Lam K.S.L., Cheung B.M.Y. (2013). High Ferritin and Low Transferrin Saturation Are Associated with Pre-Diabetes among a National Representative Sample of U.S. Adults. Clin. Nutr..

[29] Esterbauer H., Schaur R.J., Zollner H. (1991). Chemistry and Biochemistry of 4-Hydroxynonenal, Malonaldehyde and Related Aldehydes. Free Radic. Biol. Med..

[30] Rice-Evans C.A., Burdon R.H. (1994). Free Radical Damage and Its Control.

[31] Newsholme P., Krause M. (2012). Nutritional Regulation of Insulin Secretion: Implications for Diabetes. Clin. Biochem. Rev..

[32] Cooksey R.C., Jouihan H.A., Ajioka R.S., Hazel M.W., Jones D.L., Kushner J.P., McClain D.A. (2004). Oxidative Stress, Beta-Cell Apoptosis, and Decreased Insulin Secretory Capacity in Mouse Models of Hemochromatosis. Endocrinology.

[33] Kasuga M. (2006). Insulin Resistance and Pancreatic Beta Cell Failure. J. Clin. Investig..

[34] Shaaban M.A., Dawod A.E.A., Nasr M.A. (2016). Role of Iron in Diabetes Mellitus and Its Complications. Menoufia Med J..

[35] Ježek P., Jabůrek M., Plecitá-Hlavatá L. (2019). Contribution of Oxidative Stress and Impaired Biogenesis of Pancreatic β-Cells to Type 2 Diabetes. Antioxid. Redox Signal..

[36] Bao Y.-M., Sun S.-J., Li M., Li L., Cao W.-L., Luo J., Tang H.-J., Huang J., Wang Z.-F., Wang J.-F. (2012). Overexpression of the Qc-SNARE Gene OsSYP71 Enhances Tolerance to Oxidative Stress and Resistance to Rice Blast in Rice (*Oryza Sativa* L.). Gene.

[37] Munhoz A.C., Riva P., Simões D., Curi R., Carpinelli A.R. (2016). Control of Insulin Secretion by Production of Reactive Oxygen Species: Study Performed in Pancreatic Islets from Fed and 48-Hour Fasted Wistar Rats. PLoS ONE.

[38] Bock L.V., Hutchings B., Grubmüller H., Woodbury D.J. (2010). Chemomechanical Regulation of SNARE Proteins Studied with Molecular Dynamics Simulations. Biophys. J..

[39] Daraio T., Bombek L.K., Gosak M., Valladolid-Acebes I., Klemen M.S., Refai E., Berggren P.-O., Brismar K., Rupnik M.S., Bark C. (2017). SNAP-25b-Deficiency Increases Insulin Secretion and Changes Spatiotemporal Profile of Ca2 Oscillations in β Cell Networks. Sci. Rep..

[40] Aslamy A., Thurmond D.C. (2017). Exocytosis Proteins as Novel Targets for Diabetes Prevention And/or Remediation?. Am. J. Physiol. Regul. Integr. Comp. Physiol..

[41] Hou J.C., Min L., Pessin J.E. (2009). Insulin Granule Biogenesis, Trafficking and Exocytosis. Vitam. Horm..

