# Supplementary material for: Excessive Iron Induces Oxidative Stress Promoting Cellular Perturbations and Insulin Secretory Dysfunction in MIN6 Beta Cells
Source: Cells. 2021 May 9;10(5):1141. doi: 10.3390/cells10051141 (PMC8151797; doi:10.3390/cells10051141)
Supplement: Supplementary file 1 [file cells-10-01141-s001.zip › cells-1208931-supplementary.pdf]

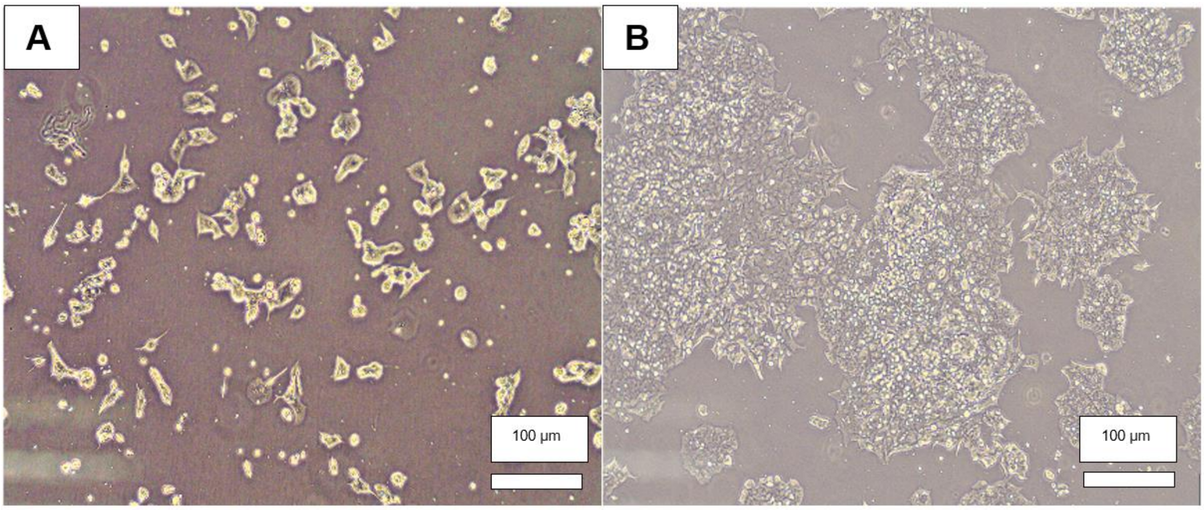

Figure S1. Growth pattern of MIN6 cells into mature pseudoislets. Stock MIN6 cell cultures were grown at 37°C in 75 cm2 T-flasks, replacing the medium every two days. Cells were seeded in 12-well plates with density of 25 x 104 cells/cm2 for all experimental cultures (panel A). MIN6 cells reached confluence on day 3 post-seeding by which point the phenotype of small clusters of cells (pseudoislets) was formed, indicating the cells were functional and capable of secreting insulin (panel B). Cells were imaged through a microscope at 10X magnification.
